# Long-Term Changes in Ovarian Follicles of Gilts Exposed Neonatally to Methoxychlor: Effects on Oocyte-Derived Factors, Anti-Müllerian Hormone, Follicle-Stimulating Hormone, and Cognate Receptors

**DOI:** 10.3390/ijms23052780

**Published:** 2022-03-03

**Authors:** Patrycja Witek, Małgorzata Grzesiak, Marek Koziorowski, Maria Slomczynska, Katarzyna Knapczyk-Stwora

**Affiliations:** 1 Department of Endocrinology, Institute of Zoology and Biomedical Research, Jagiellonian University, Gronostajowa 9, 30-387 Krakow, Poland; m.e.grzesiak@uj.edu.pl (M.G.); maria.slomczynska@uj.edu.pl (M.S.); 2 Department of Physiology and Reproduction of Animals, Institute of Biotechnology, University of Rzeszow, Werynia 502, 36-100 Kolbuszowa, Poland; mkoziorowski@ur.edu.pl

**Keywords:** oocyte-derived factors, AMH, FSH, methoxychlor, ovary, pig

## Abstract

In this paper, we investigated the effects of neonatal exposure to methoxychlor (MXC), a synthetic organochlorine used as an insecticide with estrogenic, antiestrogenic, and antiandrogenic activities on ovarian follicles of adult pigs. Piglets were injected with MXC (20 μg/kg body weight) or corn oil (controls) from postnatal Day 1 to Day 10 (*n* = 5 per group). Then, mRNA expression, protein abundance and immunolocalization of growth and differentiation factor 9 (GDF9), bone morphogenetic protein 15 (BMP15), anti-Müllerian hormone (AMH) and cognate receptors (ACVR1, BMPR1A, BMPR1B, TGFBR1, BMPR2, and AMHR2), as well as FSH receptor (FSHR) were examined in preantral and small antral ovarian follicles of sexually mature gilts. The plasma AMH and FSH levels were also assessed. In preantral follicles, neonatal exposure to MXC increased *GDF9*, *BMPR1B*, *TGFBR1*, and *BMPR2* mRNAs, while the levels of *AMH* and *BMP15* mRNAs decreased. In addition, MXC also decreased BMP15 and BMPR1B protein abundance. Regarding small antral follicles, neonatal exposure to MXC upregulated mRNAs for *BMPR1B*, *BMPR2*, and *AMHR2* and downregulated mRNAs for *AMH*, *BMPR1A*, and *FSHR*. MXC decreased the protein abundance of AMH, and all examined receptors in small antral follicles. GDF9 and BMP15 were immunolocalized in oocytes and granulosa cells of preantral follicles of control and treated ovaries. All analyzed receptors were detected in the oocytes and granulosa cells of preantral follicles, and in the granulosa and theca cells of small antral follicles. The exception, however, was FSHR, which was detected only in the granulosa cells of small antral follicles. In addition, MXC decreased the plasma AMH and FSH concentrations. In conclusion, the present study may indicate long-term effects of neonatal MXC exposure on GDF9, BMP15, AMH, and FSH signaling in ovaries of adult pigs. However, the MXC effects varied at different stages of follicular development. It seems that neonatal MXC exposure may result in accelerated initial recruitment of ovarian follicles and impaired cyclic recruitment of antral follicles.

## 1. Introduction

Methoxychlor (MXC) is a synthetic organochlorine pesticide that has replaced dichlorodiphenyltrichloroethane (DDT), which is a highly toxic albeit a potent and diversely acting insecticide [1]. Owing to toxicity and negative effects on the endocrine system, use of MXC is banned in many countries. MXC use, however, continues in developing countries [2]. Moreover, MXC tends to bioaccumulate in the environment and measurable amounts of MXC along with its metabolites are detectable in soil, water, the atmosphere, plants, and in animals including human tissues, even in regions where this chemical has not been used [3]. MXC and its metabolites can exert estrogenic, antiestrogenic, and antiandrogenic activity [4], altering functions of the reproductive system in both males and females [5,6]. MXC exposure during development has been shown to deleteriously affect folliculogenesis in rats and lead to ovulation failures and reduced fertility [7]. 

Growth and development of ovarian follicles, which provides the microenvironment for oocyte maturation, is under the regulation of bi-directional communications between oocyte and granulosa cells, and between granulosa and theca cells. Intra- and extra-follicular factors, such as steroids, gonadotropins, and growth factors regulate these processes [8,9]. For example, members of the transforming growth factor β (TGFβ) superfamily, including bone morphogenetic protein 15 (BMP15), growth differentiation factor (GDF9), and anti-Müllerian hormone (AMH), are essential in regulating oocyte development, folliculogenesis, and ovulation [10]. On the one hand, GDF9 and BMP15 are oocyte-derived factors which are implicated in the regulation of growth, differentiation and function of granulosa and theca cells during folliculogenesis, along with steroidogenesis and various processes essential for oocyte nourishment [10,11]. On the other hand, AMH, produced by the granulosa cells of growing preantral and small antral follicles, inhibits primordial follicle recruitment into the growing follicular pool, thus, preventing exhaustion of the follicular reserve. Furthermore, it has been shown that AMH modulates cyclic recruitment of small antral follicles by decreasing the responsiveness of growing follicles to follicle-stimulating hormone (FSH) in mice [12,13,14]. Biological effects of GDF9, BMP15, and AMH are the downstream consequences of binding of these factors to the serine/threonine kinase receptors type II, which induces its formation of a hetero-oligomeric complex with type I serine/threonine kinase receptors and subsequent activation of the SMAD transcription factors [10]. GDF9 activates the Smad 2/3 pathway by binding to the BMP receptor type II (BMPR2) and TGFβ receptor type I (TGFBR1) [15,16], while BMP15 activates Smad 1/5/8 signaling by binding to the BMP receptor type IB (BMPR1B) and BMPR2 [17]. AMH acts via the AMH-specific type II receptor (AMHR2), which forms a complex with one of three type I receptors, i.e., BMPR1A, BMPR1B, or activin A receptor type 1 (ACVR1), and leads to activation of the downstream signaling molecules Smad 1/5/8 [18,19].

Our previous studies showed that neonatal exposure of piglets to agonists and antagonists of sex steroid receptors affected the expression of GDF9, BMP15, and AMH, along with the expression of their receptors in ovarian follicles in adult life. Furthermore, plasma AMH and FSH concentrations showed changes [20,21]. In pigs, the neonatal period is crucial for the establishment of ovarian reserve and female reproductive potency, since formation of the primordial follicle pool is completed around postpartum Day 25 [22]. Based on these results, we hypothesize that neonatal exposure to an environmental compound with mixed steroidal properties affect folliculogenesis in adulthood. Thus, the present study aimed to characterize long-term alterations in ovarian follicles in gilts after neonatal exposure to MXC. To achieve this goal, we examined the expression of oocyte-derived factors (GDF9 and BMP15), AMH, and cognate receptors, as well as FSH receptor (FSHR), in a population of preantral and small antral ovarian follicles using real-time PCR, Western blotting, and immunohistochemistry. Plasma AMH and FSH were quantified using enzyme-linked immune sorbent assay (ELISA).

## 2. Results

### 2.1. Effect of Neonatal Exposure to MXC on GDF9 and BMP15 Expression in Preantral Follicles of Gilts

**Figure 1:**
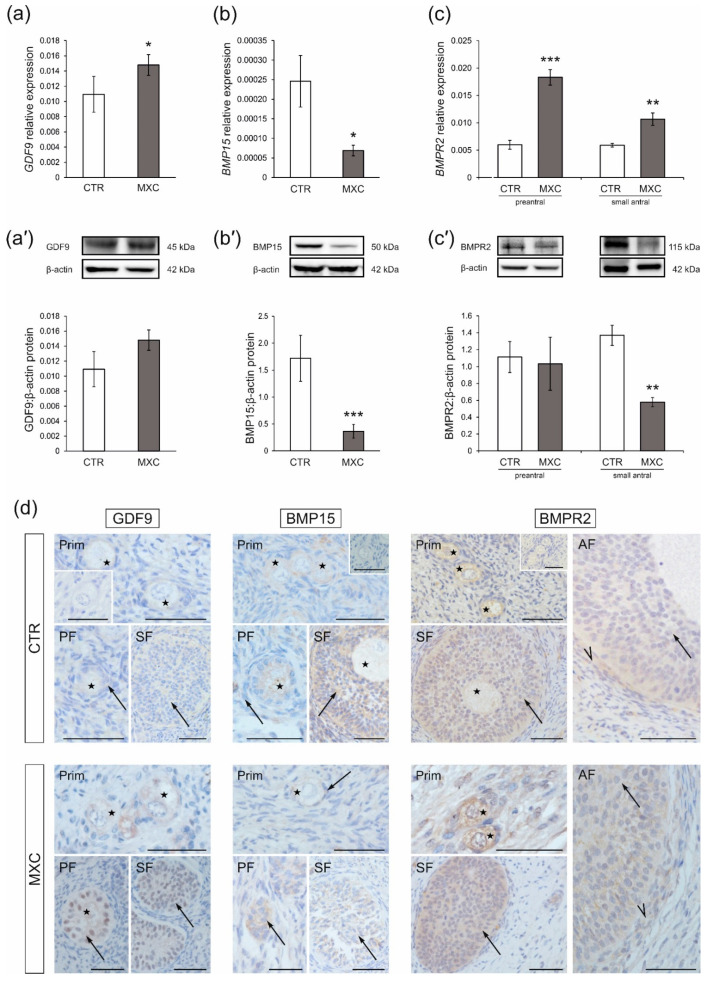
The mRNA expression and protein abundance of GDF9 (**a**,**a’**), BMP15 (**b**,**b’**) in preantral follicles, as well as BMPR2 (**c**,**c’**) in preantral and small antral follicles obtained from the control (CTR) and methoxychlor (MXC-) treated gilts. The mRNA expression (quantitative real-time PCR) is presented relative to GAPDH as mean ± SEM (**a**–**c**). Relative protein abundance was measured by the densitometric method and expressed as the ratio relative to β-actin abundance (mean ± SEM; **a’**–**c’**). The fragment of membranes with bands corresponding to predicted molecular weights is shown in the above graphs. Asterisks on the graphs denote significant differences between the CTR and MXC-treated animals (for preantral follicles pool *n* = 5, for small antral follicle *n* = 15, * *p* < 0.05, ** *p* < 0.01, *** *p* < 0.001, Mann–Whitney U test). (**d**) Localization of GDF9 and BMP15 in preantral (primordial, primary, and secondary follicle) as well as localization of BMPR2 in preantral and small antral follicles of the CTR and MXC-treated pigs. GDF9-, BMP15-, and BMPR2-positive staining was observed in oocytes (asterisks) and granulosa cells (arrows) in primordial, primary, and secondary follicles, while in small antral follicles, BMPR2-positive staining was detected in granulosa cells (arrows) and theca cells (arrowheads) in both examined groups. All sections were counterstained with hematoxylin QS. No positive staining was observed in the negative control sections (**d**, insets). Prim—primordial follicles; PF—primary follicles; SF—secondary follicles; AF—antral follicles. Bars = 50 µm.

The effects of MXC on mRNA expression and protein abundance of GDF9 and BMP15 in preantral follicles of gilts were determined using quantitative real-time PCR (Figure 1a,b) and Western blot analyses (Figure 1a’,b’). *GDF9* mRNA expression increased (Figure 1a, *p* < 0.05), while *BMP15* mRNA expression decreased in preantral follicles (Figure 1b, *p* < 0.05) of MXC-treated gilts as compared with those in the control group. In both the control and MXC-treated pigs, GDF9 and BMP15 proteins were detected in preantral follicles as bands of approximately 45 kDa (GDF9) and 50 kDa (BMP15), as shown in Figure 1a’,b’. Neonatal exposure to MXC resulted in significant downregulation of BMP15 protein abundance in the population of preantral follicles (Figure 1b’, *p* < 0.001), but no changes in GDF9 protein abundance were observed (Figure 1a’). Positive cytoplasmic GDF9 and BMP15 staining (Figure 1d) was observed, in all sections in the oocytes of primordial, primary, and secondary follicles, as well as in granulosa cells of primary and secondary follicles. Notably, while, in the control group, GDF9 positive staining was observed exclusively in the cytoplasm of granulosa cells, GDF9 was localized also in the nucleus in the MXC-treated group. Replacement of the primary antibodies with non-immune rabbit IgG was performed for the negative control sections (Figure 1d, insets).

### 2.2. Effect of Neonatal Exposure to MXC on BMPR2 Expression in Preantral and Small Antral Follicles of Gilts

The effects of neonatal MXC exposure on mRNA expression and protein abundance of BMPR2 in preantral and small antral follicles of adult pigs were demonstrated with quantitative real-time PCR (Figure 1c) and Western blot analyses (Figure 1c’). In preantral follicles and small antral follicles of both the control and MXC-treated pigs, BMPR2 was detected as bands of approximately 115 kDa (Figure 1c’). *BMPR2* mRNA expression was upregulated in both preantral (*p* < 0.001) and small antral (*p* < 0.01) follicles of gilts neonatally exposed to MXC as compared with those in the control group (Figure 1c). BMPR2 protein abundance decreased in small antral follicles (Figure 1c’, *p* < 0.01), while no changes were observed in preantral follicles after MXC exposure as compared with that in the control group (Figure 1c’). BMPR2 was localized in the oocytes and granulosa cells of preantral follicles, as well as in granulosa and theca cells of small antral follicles in the control and MXC-treated gilts (Figure 1d). The negative control sections are shown as insets in Figure 1d.

### 2.3. Effect of Neonatal Exposure to MXC on ACVR1, BMPR1A, BMPR1B, TGFBR1 Expression in Preantral and Small Antral Follicles of Gilts

**Figure 2:**
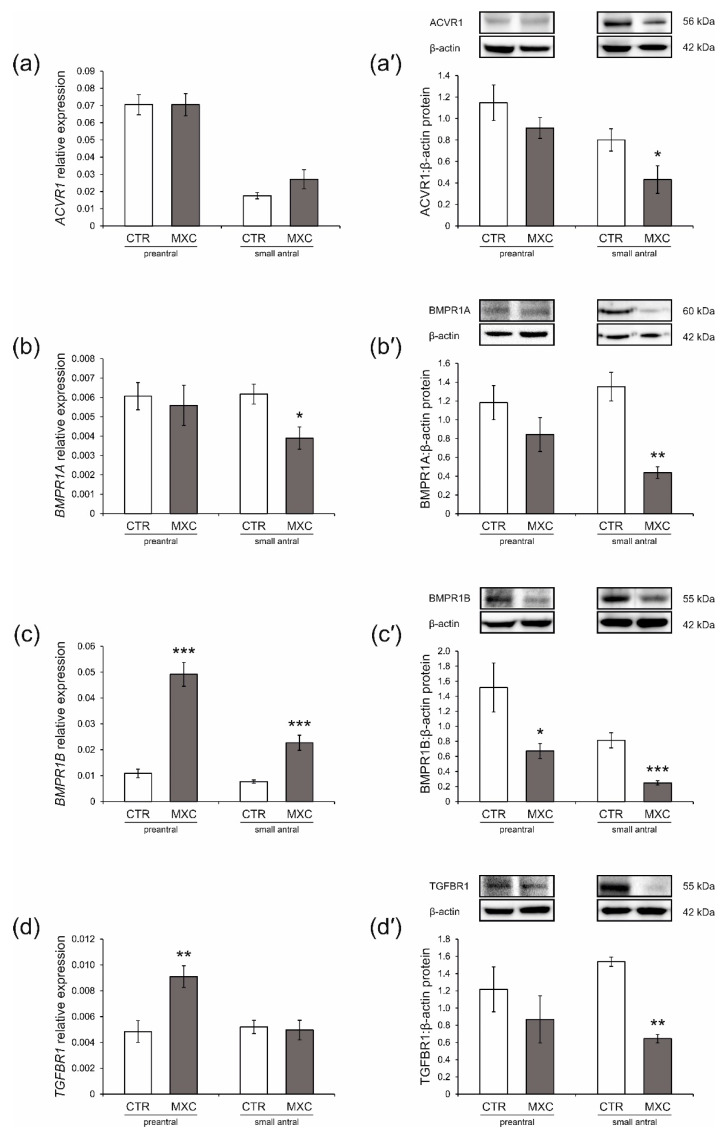
ACVR1 (**a**,**a’**), BMPR1A (**b**,**b’**), BMPR1B (**c**,**c’**), and TGFBR1 (**d**,**d’**) mRNA and protein abundance in preantral (primordial, primary, and secondary follicles) and small antral follicles obtained from the control (CTR) and methoxychlor (MXC-) treated gilts. The mRNA expression (quantitative real-time PCR) is presented relative to GAPDH as mean ± SEM (**a**–**d**). Relative protein abundance was measured by the densitometric method and expressed as the ratio relative to β-actin abundance (mean ± SEM, **a’**–**d’**). The fragment of membranes with bands corresponding to predicted molecular weights is shown above graphs. Asterisks denote significant differences between the CTR and treated animals (for preantral follicles pool *n* = 5, for small antral follicle *n* = 15, * *p* < 0.05, ** *p* < 0.01, *** *p* < 0.001, Mann–Whitney U test).

Effects of MXC on mRNA expression and protein abundance of ACVR1, BMPR1A, BMPR1B, and TGFBR1 in preantral follicles and small antral follicles were examined using quantitative real-time PCR (Figure 2a–d) and Western blot analyses (Figure 2a’–d’). In both the control and MXC-treated pigs, examined proteins were detected in preantral and small antral follicles, as shown in Figure 2a’–d’. Observed molecular weights of the analyzed proteins were approximately 56 kDa (ACVR1), 60 kDa (BMPR1A), and 55 kDa (BMPR1B and TGFBR1). MXC decreased ACVR1 protein abundance in small antral follicles as compared with that in the control group (Figure 2a’, *p* < 0.05), but no significant changes were observed in mRNA expression in both preantral and small antral follicles (Figure 2a), as well as in protein abundance in preantral follicles (Figure 2a’). *BMPR1A* mRNA expression and protein abundance were significantly lower (*p* < 0.05 and *p* < 0.01, respectively) in small antral follicles of MXC-treated gilts as compared with those in the control group (Figure 2b,b’), but no changes were observed in preantral follicles (Figure 2b,b’). *BMPR1B* mRNA expression was markedly higher in preantral and small antral follicles of MXC-treated pigs, as compared with those in the control group (Figure 2c, *p* < 0.001), while the protein abundance was lower (Figure 2c’, *p* < 0.05 and *p* < 0.001, respectively) as compared with that in the control group. MXC increased *TGFBR1* mRNA expression in preantral follicles as compared with that in the control group (Figure 2d, *p* < 0.01), but no significant changes were observed in protein abundance (Figure 2d’). MXC decreased TGFBR1 protein abundance in small antral follicles as compared with that in the control group (Figure 2d’, *p* < 0.01), but no significant changes were observed in mRNA expression (Figure 2d).

**Figure 3:**
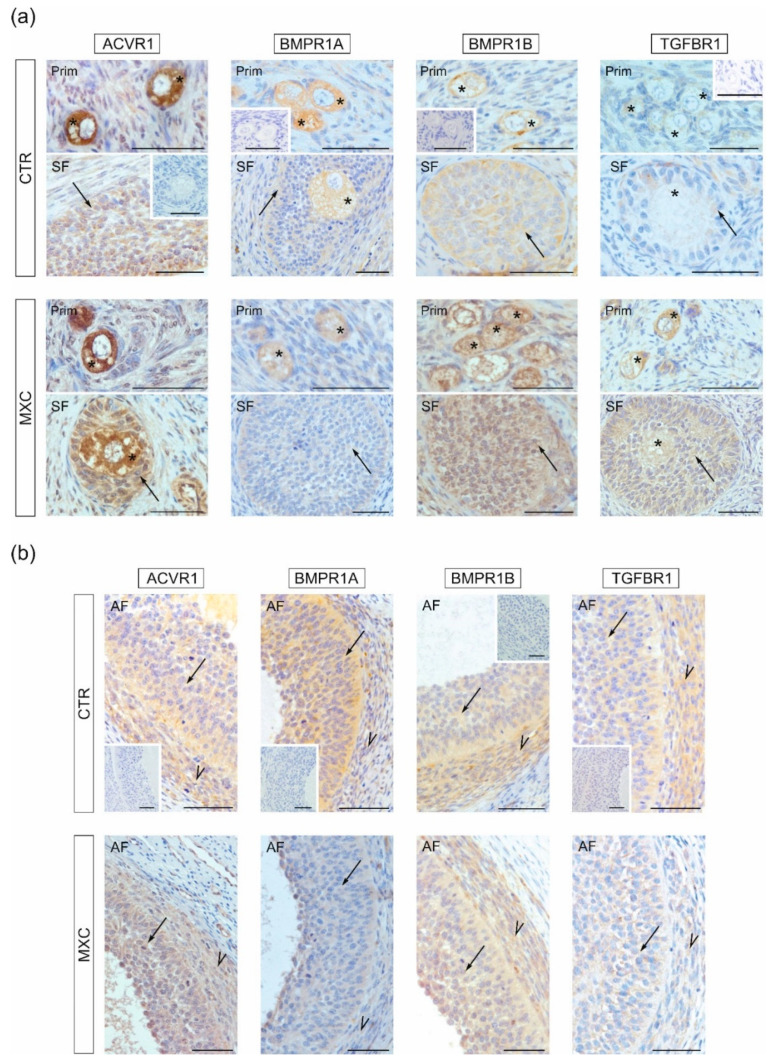
Immunolocalization of ACVR1, BMPR1A, BMPR1B, and TGFBR1 in preantral (primordial, primary, and secondary follicle) (**a**) and small antral (**b**) follicles of the control (CTR) and methoxychlor (MXC-) treated gilts. ACVR1, BMPR1A, BMPR1B, and TGFBR1 positive staining was observed in oocytes (asterisks) and granulosa cells (arrows) of preantral follicles, as well as granulosa (arrows) and theca cells (arrowheads) of small antral follicles, in both examined groups. All sections were counterstained with hematoxylin QS. There was no positive staining observed in the negative control sections (**a**,**b**, insets). Prim—primordial follicles; SF—secondary follicles; AF—antral follicles. Bars = 50 µm.

Positive immunostaining of ACVR1, BMPR1A, BMPR1B, and TGFBR1 was found in all analyzed sections. In primordial, primary, and secondary follicles ACVR1, BMPR1A, BMPR1B, and TGFBR1 were localized in oocytes and granulosa cells (Figure 3a). In small antral follicles of the control and MXC-treated groups, ACVR1, BMPR1A, BMPR1B, and TGFBR1 were observed in both granulosa and theca cells (Figure 3a). The negative control sections are shown as insets in Figure 3a,b.

### 2.4. Effect of Neonatal Exposure to MXC on Plasma AMH Concentration and the Expression of AMHR2 in Preantral and Small Antral Follicles of Gilts

**Figure 4:**
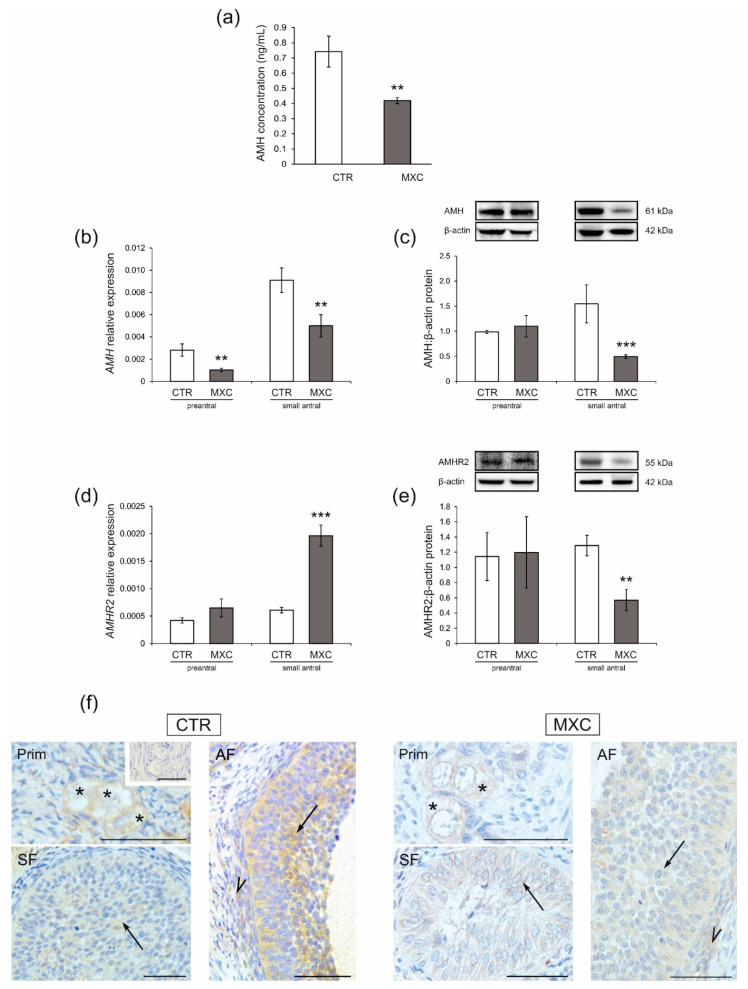
Plasma anti-Müllerian hormone (AMH) concentration in the control (CTR) and methoxychlor (MXC-) treated adult pigs (**a**) (*n* = 5 per each group). AMH (**b**,**c**) and AMHR2 (**d**,**e**) mRNA and protein abundance in preantral (primordial, primary, and secondary follicles) and small antral follicles obtained from the CTR and MXC-treated pigs (for preantral follicles pool *n* = 5, for small antral follicle *n* = 15). The mRNA expression (quantitative real-time PCR) is presented relative to GAPDH (**b**,**c**). Relative protein abundance was measured by the densitometric method and expressed as the ratio relative to β-actin abundance (**d**,**e**). Fragments of membranes with bands corresponding to predicted molecular weights are shown above graphs. Data are expressed as the mean ± SEM. Asterisks on graphs denote significant differences between the CTR and MXC-treated animals (** *p* < 0.01, *** *p* < 0.001, Mann–Whitney U test). (**f**) Localization of AMHR2 in preantral (primordial, primary, and secondary follicle) and small antral follicles of the CTR and MXC-treated pigs. AMHR2 immunopositivity was observed in oocytes (asterisks) and granulosa cells (arrows) of preantral follicles, as well as granulosa (arrows) and theca cells (arrowheads) of small antral follicles, in both examined groups. All sections were counterstained with hematoxylin QS. There was no positive staining observed in the negative control section (**f**, inset). Prim—primordial follicles; SF—secondary follicles; AF—antral follicles. Bars = 50 µm.

The plasma AMH concentration was lower in adult pigs after neonatal exposure to MXC (*p* < 0.01) as compared with that in the control group (Figure 4a). The effects of MXC on mRNA expression and protein abundance of AMH and AMHR2 in preantral follicles and small antral follicles were examined by quantitative real-time PCR (Figure 4b,d, respectively) and Western blot analyses (Figure 4c,e, respectively). In both the control and MXC-treated pigs, examined proteins were detected in preantral and small antral follicles (Figure 4c,e). The approximately observed molecular weights were: 55 kDa (AMHR2) and 61 kDa (AMH). *AMH* mRNA expression markedly decreased in both preantral and small antral follicles (Figure 4b, *p* < 0.01) of MXC-treated gilts as compared with those in the control group. AMH protein abundance decreased only in small antral follicles (Figure 4c, *p* < 0.01) as compared with that in the control group. 

MXC increased *AMHR2* mRNA expression in small antral follicles as compared with that in the control group (Figure 4d, *p* < 0.001), but protein abundance decreased (Figure 4e, *p* < 0.01). No significant changes were observed in AMHR2 expression in preantral follicles (Figure 4d,e). 

Positive cytoplasmic AMHR2 immunostaining (Figure 4f) was found, in all examined sections. AMHR2 was observed in oocytes and granulosa cells of preantral follicles, and both granulosa and theca cells of small antral follicles. The negative control section is shown as an inset in Figure 4f.

### 2.5. Effect of Neonatal Exposure to MXC on Plasma FSH Concentration and the Expression of FSHR in Preantral and Small Antral Follicles of Gilts

**Figure 5:**
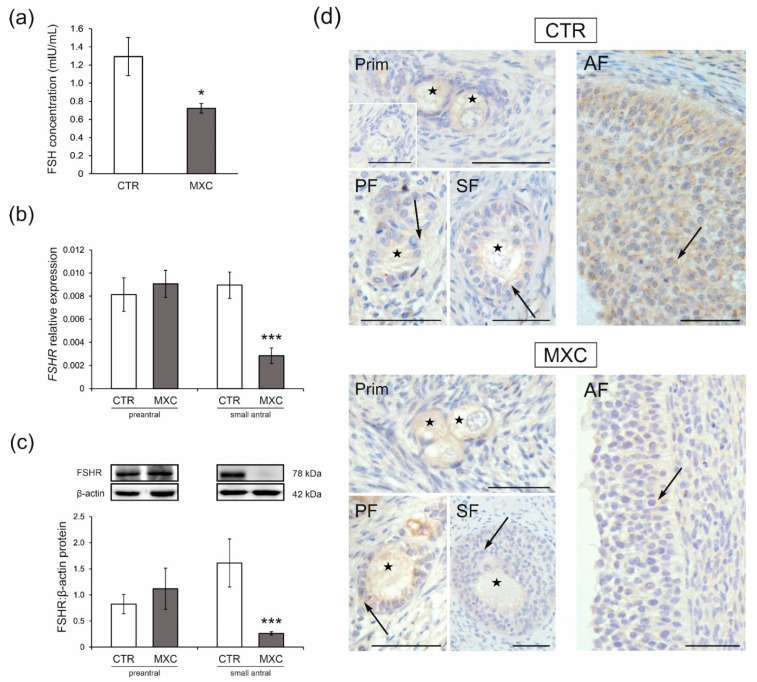
Plasma follicle-stimulating hormone (FSH) concentration in the control (CTR) and methoxychlor (MXC-) treated adult pigs (**a**) (*n* = 5 per each group). FSHR (**b**,**c**) mRNA and protein abundance in preantral (primordial, primary, and secondary follicles), as well as small antral follicles obtained from the CTR and MXC-treated gilts (for preantral follicles pool *n* = 5, for small antral follicle *n* = 15). The mRNA expression (quantitative real-time PCR) is presented relative to GAPDH (**b**). Relative protein abundance was measured by the densitometric method and expressed as the ratio relative to β-actin abundance (**c**). Membrane fragments with bands corresponding to predicted molecular weights are shown above graphs. Data are expressed as the mean ± SEM. Asterisks on graphs denote significant differences between the CTR and MXC-treated animals (* *p* < 0.05, *** *p* < 0.001, Mann–Whitney U test). (**d**) Localization of FSHR in preantral (primordial, primary, and secondary follicle) and small antral follicles of the CTR and MXC-treated pigs. FSHR immunopositivity was observed in oocytes (asterisks) and granulosa cells (arrows) of preantral follicles, as well as granulosa cells (arrows) of small antral follicles, in both examined groups. Hematoxylin QS was used for counterstaining sections. There was no positive staining observed in the negative control section (**d**, insets). Prim—primordial follicles; PF—primary follicles; SF—secondary follicles; AF—antral follicles. Bars = 50 µm.

The plasma FSH concentration decreased following neonatal exposure to MXC (*p* < 0.05) as compared with that in the control group (Figure 5a). The effects of MXC on mRNA expression and protein abundance of FSHR in preantral follicles and small antral follicles were examined by quantitative real-time PCR (Figure 5b) and Western blot analyses (Figure 5c). In both the control and MXC-treated pigs, examined proteins were detected in preantral and small antral follicles, as shown in Figure 5c. The observed FSHR molecular weight was approximately 78 kDa. No statistically significant changes in *FSHR* mRNAs (Figure 5b) and protein abundance (Figure 5c) were observed in the population of preantral follicles as compared with those in the control group. *FSHR* mRNA expression (Figure 5b) and protein abundance (Figure 5c) were downregulated in small antral follicles (*p* < 0.001) of MXC-treated gilts as compared with those in the control group. Positive FSHR immunostaining (Figure 5d) was observed in the oocytes and granulosa cells of preantral and small antral follicles, in all examined sections. The negative control section is shown as an inset in Figure 5d.

## 3. Discussion

Recently, we have reported long-term effects of neonatal MXC exposure on the transcriptome of luteal tissue in gilts. Our data suggest an earlier onset of structural luteolysis and a crucial role of steroid milieu in ovarian development during the neonatal window [23]. We have also found that neonatal exposure of piglets to agonists or antagonists of sex steroid receptors, including environmental estrogen 4-tert-octylphenol, influenced the action of intra-ovarian factors including BMP15, GDF9, and AMH, which suggested impaired initial ovarian follicle recruitment [20,21]. To extend this line of research, in the present study, we investigated the long-term effects of MXC on the expression of oocyte-derived factors and their receptors, as well as on AMH and FSH signaling in ovarian follicles of adult pigs. 

Oocyte-derived GDF9 and BMP15 are essential in the initial recruitment of follicles and in their development to the preantral stage [16,24]. The results of the current study show that neonatal exposure to MXC elevated GDF9 mRNAs, while reducing BMP15 mRNAs and protein abundance in preantral follicles of gilts. In vivo or in vitro studies in rats and buffalo have shown that GDF9 treatment enhanced progression of primordial and primary follicles into small preantral follicles [25,26]. Our previous research demonstrated increased GDF9 expression in preantral follicles of gilts exposed neonatally to androgen or antiandrogen [20]. Therefore, it seems that elevated GDF9 mRNAs in preantral follicles of gilts exposed neonatally to MXC is due to its antiandrogenic property which may accelerate initial follicle recruitment. However, no changes in GDF9 protein abundance were observed. In addition, depletion of BMP15 has been shown to disrupt ovarian function and female fertility [27]. Since BMP15 is also responsible for early follicle development [24], diminished BMP15 expression in preantral follicles of MXC-treated gilts, observed herein, may be an additional factor responsible for disrupted folliculogenesis. 

Synergistic cooperation of GDF9 and BMP15 heterodimers and homodimers within ovarian follicles has been reported [10]. GDF9 and BMP15 act via the same type II receptor, i.e., BMPR2, but different type I receptors. TGFBR1 is a type I receptor for GDF9 [15,16], while BMPR1B is associated with BMP15 action [17]. In the current study, expressions of TGFBR1, BMPR1B, and BMPR2 were upregulated in preantral follicles of gilts after neonatal MXC exposure, but only at the mRNA level. Changes at the protein level were observed only for BMPR1B, for which protein abundance was lower in preantral follicles of MXC-treated gilts. It is noteworthy that no correlations between gene and protein expression levels were observed, not only for GDF9 and BMP15 receptors. These findings are not surprising in light of studies by others that demonstrated low correlation between protein and mRNA concentrations in multicellular organisms [28,29]. Moreover, it was shown that the correlations are particularly poor for genes of signal transduction and transcriptional regulation [28]. It may be due to various biological factors, including processes regulated by post-transcriptional, translational, and protein degradation mechanisms [29]. The impact of MXC may involve post-transcriptional gene regulation such as via microRNA (miRNA) action, since we have observed changes in the expression of specific miRNAs in luteal tissue in response to neonatal MXC exposure. These results suggested miRNAs as the potential mediators of the long-term MXC effect on luteal function [23]. Notably, Yi et al. [30] reported that defective BMPR1B led to irregular estrous cycles and defects in cumulus expansion, which made fertilization impossible. Since BMPR1B is a type I BMP15 receptor, it is likely that follicle maturation and fertility in adult pigs are affected by diminished protein abundance of both BMP15 and BMPR1B in preantral follicles after neonatal MXC exposure.

AMH, which is expressed in granulosa cells of growing follicles, is another regulator of initial follicle recruitment owing to its inhibition of primordial follicle recruitment into the growing pool [31]. Uzumcu et al. [32] demonstrated that, in rats, neonatal MXC exposure inhibited ovarian follicle development along with a reduction in antral follicles number. Indeed, our previous study on neonatal ovary showed that MXC treatment decreased the percentage of developing follicles in piglets [33]. In the present study, we observed reduced plasma AMH and *AMH* mRNAs in preantral follicles of gilts exposed to MXC in neonatal life. AMH exerts its biological function by binding to AMHR2 which dimerizes with ACVR1, BMPR1A, or BMPR1B [18,19]. We observed no changes in the expressions of ACVR1, BMPR1A, and AMHR2 in preantral follicles in response to neonatal treatment with MXC; only BMPR1B protein abundance was downregulated, as previously mentioned. Considering that BMPR1B is a type I receptor for both BMP15 and AMH, downregulation of BMPR1B and BMP15 in preantral follicles of gilts and diminished plasma AMH upon neonatal MXC exposure may alter AMH and BMP15 signaling. This, in turn, would disrupt maturation of ovarian follicles and accelerate initial follicle recruitment, thereby, causing premature depletion of ovarian follicles.

Although initial recruitment and development of preantral follicles is gonadotropin independent, FSH can accelerate the onset of primordial follicle growth [31]. Studies in mice have shown that secondary follicles need sufficient FSH to develop and survive [34], and AMH reduced preantral follicles responsiveness to FSH, thereby, inhibiting follicle growth [35]. Furthermore, reduced plasma FSH has been observed in MXC-treated rats and mice [36,37]. In our current study with gilts, neonatal exposure to MXC decreased plasma FSH, which may lead to impaired development and survival of secondary follicles. Our previous study showed that neonatal treatment with androgenic and antiandrogenic compounds diminished plasma AMH and FSH [21]. Therefore, it appears that, as in the case for GDF9, MXC’s long-term effects on AMH and FSH levels may be due to its antiandrogenic property, which may have the greatest impact on preantral follicles growth in the adult pig. Indeed, androgens are more important than estrogens at early stages of folliculogenesis [38].

However, estrogens are crucial for the FSH-dependent maturation of follicles beyond the preantral stage [39]. Therefore, with its mixed steroidal properties, i.e., estrogenic, antiestrogenic, and/or antiandrogenic, MXC may have detrimental effects not only on preantral follicle development but also on the development and maturation of antral follicles. GDF9 and BMP15 have been shown to control the maturation and function of granulosa and theca cells, which are crucial for steroidogenesis, oocyte maturation, ovulation, and luteolysis [10]. Jayawardana et al. [40], suggested that GDF9 was linked to a process of antral follicle selection. In addition, estradiol was found to upregulate *TGFBR1* and *BMPR2* mRNAs in bovine granulosa cells. Our recent study in the gilt model showed that neonatal exposure to an estrogenic compound increased TGFBR1 and BMPR2 expression and decreased BMPR1B expression, while antiandrogen exposure upregulated BMPR1B and BMPR2 and downregulated TGFBR1 in small antral follicles [20]. In the present study, neonatal MXC exposure resulted in higher *BMPR2* and *BMPR1B* mRNAs, but lower protein abundance for BMPR2, BMPR1B, and TGFBR1 in small antral follicles. These findings confirm that hormonally active compounds may affect GDF9 and BMP15 signaling in antral follicles and suggest that neonatal MXC exposure may have a long-term effect on the development and function of small antral follicles in adult pigs.

Survival and differentiation of early antral follicles depend on FSH, which is involved in follicular growth, maturation, and selection of dominant follicles and also, in estradiol production [41]. Moreover, upon stimulated by FSH, AMH inhibits estradiol production and diminishes antral follicles responsiveness to FSH [42]. In the current study, neonatal treatment with MXC decreased *AMH*, *BMPR1A*, and *FSHR* mRNAs, and increased *AMHR2* and *BMPR1B* mRNAs in small antral follicles of adult pigs. However, the protein abundance of AMH, BMPR1s, AMHR2, and FSHR decreased after neonatal exposure to MXC. Moreover, as mentioned above, AMH and FSH plasma concentrations were lower in adult pigs exposed neonatally to MXC. These findings suggest that MXC has an impact on the cyclic recruitment of ovarian follicles, and it may impair estradiol synthesis. Similar results were observed in our previous study, where neonatal exposure to antiandrogenic, estrogenic, or antiestrogenic compounds altered the AMH and FSH signaling in small antral follicles of adult pigs, which possibly could lead to disruption in the maturation of ovarian follicles [21]. Early-life exposure to environmental toxicants may result in ovarian reprogramming with consequences that could be observed in adulthood [43]. Thus, our results confirm that neonatal exposure to the environmental toxicant MXC may have long-term detrimental effects not only on small antral follicle development, but also on the pituitary-ovarian axis.

In the current study, we show that GDF9 and BMP15, their cognate receptors, as well as AMH and FSH receptors are localized in oocytes and granulosa cells of preantral follicles in the control and MXC-treated gilts. This is consistent with the data in the literature and our earlier research [20,21,44]. Notably, in the control group, GDF9 is localized only in the cytoplasm of granulosa cells, while in follicles of pigs exposed neonatally to MXC, GDF9 is localized both in the cytoplasm and nuclei of granulosa cells of primary and secondary follicles. A similar pattern of staining was observed in our previous study, where neonatal treatment with an agonist or antagonist of androgen and estrogen receptors also changed localization of GDF9 in preantral follicles of adult pigs [20]. Although GDF9 nuclear expression has been observed in normal and cancerous human renal tubular epithelial cells suggesting that intracellular redistribution of GDF9 may be involved in cancer progression [45,46], data concerning nuclear GDF9 expression are limited, and it needs further research. Moreover, in small antral follicles obtained from the control and MXC-treated groups, receptors for GDF9, BMP15, and AMH were detected in granulosa and theca cells, while FSHR was detected only in granulosa cells. Our findings are consistent with the data in the literature that show localization of these receptors in the porcine ovary [20,21,44,47]. Our results show that neonatal exposure to MXC has no impact on the localization of examined proteins in preantral and small antral follicles of adult pigs.

## 4. Materials and Methods

### 4.1. Animals and Tissue Preparation

The animal care and experiments were consistent with national guidelines and were followed by the guidelines of the Local Ethics Committee at the Jagiellonian University in Krakow, Poland (permit numbers 150/2013 and 123/2014). Ten sexually mature gilts at 10–11 months of age (Large White × Polish Landrace) were used. During the neonatal period, piglets were randomly divided into two groups and injected with MXC (Sigma-Aldrich, St. Louis, MO, USA) at 20 μg/kg body weight (*n* = 5) or vehicle only (corn oil) (CTR, *n* = 5). Animals received daily subcutaneous injections from postnatal Day 1 to Day 10. The dose of MXC was based on data in the literature [7]. Animals were maintained until sexual maturity and, following two estrous cycles, animals were slaughtered at a local abattoir for ovaries collection between Day 9 and Day 10 of the estrous cycle. Before slaughtering, blood was collected from the jugular vein, and then plasma fraction was centrifugated at 2000× *g* for 10 min at 4 °C. Following excision, the ovaries were transported to the laboratory in ice-cold phosphate-buffered saline ((PBS) pH 7.4, PAA The Cell Culture Company, Piscataway, NJ, USA) containing antibiotic/antimycotic solution (AAS 10 µl/mL, PAA The Cell Culture Company, Piscataway, NJ, USA). Small antral follicles (2–4 mm) were dissected from the ovary, fixed in Bouin’s solution for immunohistochemistry or bisected, and snap-frozen in liquid nitrogen for RNA or protein isolation (*n* = 15 per each group, 3 small antral follicles per each animal). The population of preantral follicles (primordial, primary, and early secondary) were enzymatically digested from the ovarian cortex, as previously described [20]. Concisely, each ovary was cut longitudinally in halves and the medulla was removed. After rinsing of ovarian cortexes in Dulbecco’s PBS medium (PAA The Cell Culture Company, Piscataway, NJ, USA) uniform-sized pieces measuring 1 × 1 × 1 mm were obtained using a tissue slicer (Tissue Slicer Coronal, World Precision Instruments, Sarasota, FL, USA). Next, the fragments were placed in digestion medium: 10 mL of PBS, enriched with 0.08 mg/mL Liberase TH (Thermolysin High, Sigma-Aldrich, St. Louis, MO, USA) and 0.2 mg/mL DNase (Sigma-Aldrich, St. Louis, MO, USA), and incubated at 37 °C with gentle agitation for 120 min and additional manual pipetting every 30 min to release the follicles from the stroma. Enzymatic digestion was stopped by the addition of an equal volume of cold (4 °C) PBS supplemented with 10% fetal bovine serum (Sigma-Aldrich, St. Louis, MO, USA). Afterward, the digested ovarian cortex was filtered through nylon filters (Greiner Bio-One GmbH, Frickenhausen, Germany) with a filter of pore size of 70 µm, and then 40 µm. The flow-through was centrifuged at 5000× *g* for 10 min at 4 °C. The pellet was snap-frozen in liquid nitrogen for RNA or protein isolation (*n* = 5 per each group). Sections of the cortex from each ovary were fixed in Bouin’s solution for immunohistochemistry.

### 4.2. Hormone Assays

The plasma AMH and FSH concentrations (*n* = 5 per each group) were measured by ELISA using a pig Mullerian-inhibiting factor ELISA kit (cat. no. E0228p Wuhan EIAab Science Co., Ltd., Wuhan, China) and a pig follitropin subunit beta ELISA kit (cat. no. E0830p, Wuhan EIAab Science Co., Ltd.), following the manufacturer’s instructions. The assay sensitivity was 0.091 ng/mL for AMH and 0.39 mIU/ml for FSH. The intra-assay CV was ≤5.6% and 7.2% and the inter-assay CV was ≤7.8% and 10.1% for AMH and FSH, respectively. All samples were assayed in duplicate, and mean values were used for further evaluations.

### 4.3. Real-Time PCR

Total RNA was isolated from the population of preantral follicles (*n* = 5 per each group) and small antral follicles (*n* = 15, 3 small antral follicles per each animal) using TRI Reagent solution (Ambion, Austin, TX, USA), in accordance with the protocol of the manufacturer. The concentration and quality of extracted RNAs were determined using a NanoDrop ND2000 Spectrophotometer (Thermo Scientific, Wilmington, DE, USA). One microgram of total RNA was reverse transcribed to cDNA using a high-capacity cDNA reverse transcription kit (Applied Biosystems, Foster City, CA, USA). Real-time quantitative PCR (qPCR) of cDNAs was performed using TaqMan Gene Expression Master Mix (Applied Biosystems) and porcine-specific TaqMan Gene Expression Assay (Applied Biosystems) for: *AMH* (assay ID: Ss03383931_m1), *AMHR2* (assay ID: Ss04321772_m1), *BMP15* (assay ID: Ss04248749_s1), *BMPR1A* (assay ID: Ss04248558_m1), *BMPR1B* (assay ID: Ss03380019_u1), *BMPR2* (assay ID: Ss04248598_m1), *FSHR* (assay ID: Ss03384581_u1), *GDF9* (assay ID: Ss03391680_m1), *TGFBR1* (assay ID: Ss03392139_m1), and glyceraldehyde-3-phosphate dehydrogenase ((*GAPDH*) assay ID: Ss03373286_u1) as endogenous control, following the manufacturers’ instructions. PCR amplifications were run on a StepOne Real-Time PCR System (Applied Biosystems). *ACVR1* mRNA expression was measured using primers established based on the gene sequences found in the Ensembl database utilizing the Primer3 software (http://bioinfo.ut.ee/primer3/, accessed on 8 February 2022) and the analysis was conducted with SYBR Green master mix (Applied Biosystems). Amplifications were implemented using the StepOne Real-Time PCR System, according to the cycling conditions recommended by the manufacturer, including post-amplification melting curve analysis (ramp +0.5 °C) to confirm the absence of primer dimmers. Primers used were as follows: forward, 5′-CATCAGCTTAGCCAGAGAGGTT-3′ and reverse, 5′-AGGTGGATTGCTTCGATTCTTA-3′ for *ACVR1*; forward, 5′-TGCTGTAGCCAAATTCATTGTC-3′ and reverse, 5′-GATGACATCAAGAAGGTGGTGA-3′, for *GAPDH*. Genomic DNA amplification contamination was checked by control experiments based on omitting reverse transcriptase during the reverse transcription step. Each sample was run in duplicate together with a non-template control. Relative mRNA quantification was done using the real-time PCR Miner algorithm [48] and *GAPDH* for normalization. 

### 4.4. Western Blot

Extraction of proteins from the population of preantral follicles (*n* = 5 per each group) and small antral follicles (*n* = 15, 3 small antral follicles per each animal) and Western blot analysis were carried out as before [20]. Briefly, equal amounts of isolated proteins (20 µg) were resolved by electrophoresis under reducing conditions on 12% sodium dodecyl sulfate-polyacrylamide gel [49] and electroblotted onto poly(vinylidene fluoride) (PVDF) membranes. Blotted membranes were blocked for non-specific binding sites upon incubation with Tris-buffered saline (0.05 M Tris-HCl, pH 7.4) + 0.2% Tween 20 (TBST) containing 5% (*v*/*v*) non-fat dry milk for 1 h (room temperature, with shaking), followed by overnight incubation at 4 °C with primary antibodies (the antibodies and their suitable dilutions are listed in Table 1). Next, the membranes were incubated for 1 h at room temperature with secondary anti-rabbit (in the case of AMH, AMHR2, BMP15, BMPR1A, BMPR1B, BMPR2, FSHR, GDF9, and TGFBR1) or anti-goat (for ACVR1) antibodies linked to horseradish peroxidase (Jackson ImmunoResearch, Cambridge, UK) at 1:10000 antibody dilution. The antibody–antigen binding sites were detected with chemiluminescence by Western Bright Quantum substrate (Advansta, Menlo Park, CA, USA) and visualized using ChemiDoc XRS + System (Bio-Rad Labs, GmbH, Munchen, Germany). Afterward, each membrane was stripped and reprobed with monoclonal mouse anti-β-actin antibody (1:3000, Sigma-Aldrich) followed by horseradish peroxidase-conjugated anti-mouse IgG (1:10000, Jackson ImmunoResearch). Bands were quantitated by the densitometric method using the ImageJ software (National Institutes of Health, Bethesda, MD, USA) and normalized to corresponding β-actin protein abundance. 

### 4.5. Immunohistochemistry

Immunohistochemistry was conducted as before [20,21]. The blocking step prior to incubation with primary antibodies was performed with either 5% (*v*/*v*) normal goat serum (in case of AMHR2, BMP15, BMPR1A, BMPR1B, BMPR2, FSHR, GDF9, and TGFBR1) or 5% (*v*/*v*) horse serum (for ACVR1). Following overnight incubation at 4 °C with primary antibodies (the antibodies and their suitable dilutions are listed in Table 1), the sections were incubated with respective biotinylated secondary antibodies (anti-rabbit or anti-goat IgGs, Vector Laboratories, Burlingame, CA, USA) at 1:300 dilution for 1.5 h at room temperature. Next, sections were incubated with avidin-biotinylated horseradish peroxidase complex (ABC/HRP, Dako, Glostrup, Denmark) at 1:100 dilution for 40 min at room temperature. The antigen–antibody complex was visualized with 3,3´-diaminobenzidine (DAB, Sigma-Aldrich, St. Louis, MO, USA) as a chromogen staining substrate. The sections were counterstained with hematoxylin QS (Vector Laboratories, Burlingame, CA, USA). All negative control sections were obtained by performing a parallel staining by replacing the primary with non-immune rabbit or goat IgG to ensure the absence of non-specific staining. Tissue sections (*n* = 5 per each group) were analyzed under light microscopy (Nikon Eclipse Ni-U microscope) and photographed with a Nikon Digital DS-Fi1-U3 camera (Nikon, Tokyo, Japan) with the corresponding software.

### 4.6. Statistical Analysis

Statistical analysis was performed using the Statistica v.13.1 program (StatSoft, Inc., Tulsa, OK, USA). The data are all shown as the mean ± SEM. Due to lack of normal distribution, the nonparametric Mann–Whitney U test was used to determine significant differences between the control and MXC-treated groups. Differences were considered statistically significant at *p* < 0.05.

## 5. Conclusions

The current study confirms that proper ovarian function in adulthood is programmed during the neonatal window. Neonatal exposure to MXC affected GDF9 and BMP15 signaling in adult porcine ovaries. However, the MXC effects varied among different stages of follicular development, showing more prominent changes for BMP15 signaling in small antral follicles. Our findings suggest that neonatal MXC exposure renders long-term effects that may result in accelerated initial recruitment of ovarian follicles leading to premature ovarian failure. Moreover, neonatal MXC treatment in gilts reduced plasma AMH and FSH levels, which further indicated a possibly accelerated primordial follicle recruitment and impaired cyclic recruitment of antral follicles. It seems that the mixed steroidal activity of MXC is the primary cause of its detrimental effects on the development of both preantral and small antral follicle. Since environmental pollutants with endocrine disruptive effects are extensively used in modern living, their long-term effects on female reproduction and fertility should be thoroughly investigated.

## Figures and Tables

**Table 1:** List of primary antibodies used for Western blot and immunohistochemistry.

Antibody	Dilution Used for		Species	Supplier
	WB	IHC		
Anti-ACVR1	1:1000	1:200	goat polyclonal	Acris Antibodies GmbH, Herford, Germany (AP22507PU-N, Lot no. 19042)
Anti-AMH	1:1000	-	rabbit polyclonal	Acris Antibodies GmbH, Herford, Germany (TA336233, Lot no. OC22813)
Anti-AMHR2	1:1000	1:100	rabbit polyclonal	LifeSpan BioSciences Inc., Seattle, WA, USA (LS-B11943, Lot no. 44994)
Anti-BMP15	1:500	1:50	rabbit polyclonal	Biorbyt Ltd., Cambridge, UK (orb377952, Lot no. CQ2185)
Anti-BMPR1A	1:1000	1:100	rabbit polyclonal	Kindly provided by Prof. C. H. Heldin (Ludwig Institute for Cancer Research Ltd., Uppsala, Sweden)
Anti-BMPR1B	1:1000	1:100	rabbit polyclonal	Kindly provided by Prof. C. H. Heldin (Ludwig Institute for Cancer Research Ltd., Uppsala, Sweden)
Anti-BMPR2	1:1000	1:100	rabbit polyclonal	Kindly provided by Prof. C. H. Heldin (Ludwig Institute for Cancer Research Ltd., Uppsala, Sweden)
Anti-FSHR	1:1000	1:100	rabbit polyclonal	Bioss Antibodies, Woburn, MA, USA (BS-0895R, Lot no. AF12207065)
Anti-GDF9	1:1000	1:500	rabbit polyclonal	Abcam Cambridge, UK (ab93892, Lot no. GR269496-1)
Anti-TGFBR1	1:1000	1:50	rabbit polyclonal	Abgent San Diego, CA, USA (AP7822c, Lot no. SA110808BQ)

Abbreviations: ACVR1—activin A receptor type 1; AMH—anti-Müllerian hormone; AMHR2—anti-Müllerian hormone type II receptor; BMP15—bone morphogenetic protein 15; BMPR1A—bone morphogenetic protein receptor type 1A; BMPR1B—bone morphogenetic protein receptor type 1B; BMPR2—bone morphogenetic protein receptor type 2; FSHR—follicle-stimulating hormone receptor; GDF9—growth differentiation factor 9; TGFBR1—transforming growth factor beta receptor 1; IHC—immunohistochemistry; WB—Western blot.

## Data Availability

Data available from the corresponding author on reasonable request.

## References

[1] Hwang K.-A., Choi K.-C. (2015). Endocrine-disrupting chemicals with estrogenicity posing the risk of cancer progression in estrogen-responsive organs. Adv. Mol. Toxicol..

[2] WHO (World Health Organization) (2011). Global Insecticide Use for Vector-Borne Disease Control: A 10-Year Assessment, 2000–2009.

[3] Persistent Organic Pollutants Review Committee Methoxychlor: Draft Risk Profile. https://echa.europa.eu/documents/10162/b65a738e-b50f-64e5-cbb0-f2711d49c25e.

[4] Gaido K.W., Maness S.C., McDonnell D.P., Dehal S.S., Kupfer D., Safe S. (2000). Interaction of methoxychlor and related compounds with estrogen receptor alpha and beta, and androgen receptor: Structure-activity studies. Mol. Pharmacol..

[5] Amstislavsky S.Y., Amstislavskaya T.G., Amstislavsky V.S., Tibeikina M.A., Osipov K.V., Eroschenko V.P. (2006). Reproductive abnormalities in adult male mice following preimplantation exposures to estradiol or pesticide methoxychlor. Reprod. Toxicol..

[6] Patel S., Zhou C., Rattan S., Flaws J.A. (2015). Effects of endocrine-disrupting chemicals on the Ovary. Biol. Reprod..

[7] Armenti A.E., Zama A.M., Passantino L., Uzumcu M. (2008). Developmental methoxychlor exposure affects multiple reproductive parameters and ovarian folliculogenesis and gene expression in adult rats. Toxicol. Appl. Pharmacol..

[8] Eppig J.J. (2001). Oocyte control of ovarian follicular development and function in mammals. Reproduction.

[9] Kidder G.M., Vanderhyden B.C. (2010). Bidirectional communication between oocytes and follicle cells: Ensuring oocyte developmental competence. Can. J. Physiol. Pharmacol..

[10] Sanfins A., Rodrigues P., Albertini D.F. (2018). GDF-9 and BMP-15 direct the follicle symphony. J. Assist. Reprod. Genet..

[11] De Castro F.C., Cruz M.H., Leal C.L. (2016). Role of growth differentiation factor 9 and bone morphogenetic protein 15 in ovarian function and their importance in mammalian female fertility—A review. Asian-Australas. J. Anim. Sci..

[12] Durlinger A.L., Gruijters M.J., Kramer P., Karels B., Kumar T.R., Matzuk M.M., Rose U.M., de Jong F.H., Uilenbroek J.T., Grootegoed J.A. (2001). Anti-Müllerian hormone attenuates the effects of FSH on follicle development in the mouse ovary. Endocrinology.

[13] Durlinger A.L., Gruijters M.J., Kramer P., Karels B., Ingraham H.A., Nachtigal M.W., Uilenbroek J.T., Grootegoed J.A., Themmen A.P. (2002). Anti-Müllerian hormone inhibits initiation of primordial follicle growth in the mouse ovary. Endocrinology.

[14] Salmon N.A., Handyside A.H., Joyce I.M. (2004). Oocyte regulation of anti-Müllerian hormone expression in granulosa cells during ovarian follicle development in mice. Dev. Biol..

[15] Vitt U.A., Mazerbourg S., Klein C., Hsueh A.J. (2002). Bone morphogenetic protein receptor type II is a receptor for growth differentiation factor-9. Biol. Reprod..

[16] Mazerbourg S., Klein C., Roh J., Kaivo-Oja N., Mottershead D.G., Korchynskyi O., Ritvos O., Hsueh A.J. (2004). Growth differentiation factor-9 signaling is mediated by the type I receptor, activin receptor-like kinase 5. Mol. Endocrinol..

[17] Peng J., Li Q., Wigglesworth K., Rangarajan A., Kattamuri C., Peterson R.T., Eppig J.J., Thompson T.B., Matzuk M.M. (2013). Growth differentiation factor 9:bone morphogenetic protein 15 heterodimers are potent regulators of ovarian functions. Proc. Natl. Acad. Sci. USA.

[18] Di Clemente N., Josso N., Gouédard L., Belville C. (2003). Components of the anti-Müllerian hormone signaling pathway in gonads. Mol. Cell. Endocrinol..

[19] Gruijters M.J., Visser J.A., Durlinger A.L., Themmen A.P. (2003). Anti-Müllerian hormone and its role in ovarian function. Mol. Cell. Endocrinol..

[20] Knapczyk-Stwora K., Grzesiak M., Witek P., Duda M., Koziorowski M., Slomczynska M. (2019). Neonatal exposure to agonists and antagonists of sex steroid receptors induces changes in the expression of oocyte-derived growth factors and their receptors in ovarian follicles in gilts. Theriogenology.

[21] Knapczyk-Stwora K., Grzesiak M., Witek P., Duda M., Koziorowski M., Slomczynska M. (2020). Neonatal exposure to agonists and antagonists of sex steroid receptors affects AMH and FSH plasma level and their receptors expression in the adult pig ovary. Animals.

[22] Monniaux D., Clément F., Dalbiès-Tran R., Estienne A., Fabre S., Mansanet C., Monget P. (2014). The ovarian reserve of primordial follicles and the dynamic reserve of antral growing follicles: What is the link?. Biol. Reprod..

[23] Witek P., Enguita F.J., Grzesiak M., Costa M.C., Gabriel A., Koziorowski M., Slomczynska M., Knapczyk-Stwora K. (2021). Effects of neonatal exposure to methoxychlor on corpus luteum in gilts: A transcriptomic analysis. Mol. Reprod. Dev..

[24] Otsuka F., McTavish K.J., Shimasaki S. (2011). Integral role of GDF-9 and BMP-15 in ovarian function. Mol. Reprod. Dev..

[25] Vitt U.A., McGee E.A., Hayashi M., Hsueh A.J. (2000). In vivo treatment with GDF-9 stimulates primordial and primary follicle progression and theca cell marker CYP17 in ovaries of immature rats. Endocrinology.

[26] Abdel-Ghani M.A., El-Sherry T.M., Abdelhafeez H.H. (2016). Effect of growth differentiation factor-9 (GDF-9) on the progression of buffalo follicles in vitrified-warmed ovarian tissues. Reprod. Domest. Anim..

[27] Patiño L.C., Walton K.L., Mueller T.D., Johnson K.E., Stocker W., Richani D., Agapiou D., Gilchrist R.B., Laissue P., Harrison C.A. (2017). BMP15 mutations associated with Primary Ovarian Insufficiency reduce expression, activity, or synergy with GDF9. J. Clin. Endocrinol. Metab..

[28] De Sousa Abreu R., Penalva L.O., Marcotte E.M., Vogel C. (2009). Global signatures of protein and mRNA expression levels. Mol. Biosyst..

[29] Vogel C., Marcotte E.M. (2012). Insights into the regulation of protein abundance from proteomic and transcriptomic analyses. Nat. Rev. Genet..

[30] Yi S.E., LaPolt P.S., Yoon B.S., Chen J.Y., Lu J.K., Lyons K.M. (2001). The type I BMP receptor BmprIB is essential for female reproductive function. Proc. Natl. Acad. Sci. USA.

[31] Durlinger A.L., Kramer P., Karels B., de Jong F.H., Uilenbroek J.T., Grootegoed J.A., Themmen A.P. (1999). Control of primordial follicle recruitment by anti-Müllerian hormone in the mouse ovary. Endocrinology.

[32] Uzumcu M., Kuhn P.E., Marano J.E., Armenti A.E., Passantino L. (2006). Early postnatal methoxychlor exposure inhibits folliculogenesis and stimulates anti-Mullerian hormone production in the rat ovary. J. Endocrinol..

[33] Knapczyk-Stwora K., Grzesiak M., Ciereszko R.E., Czaja E., Koziorowski M., Slomczynska M. (2018). The impact of sex steroid agonists and antagonists on folliculogenesis in the neonatal porcine ovary via cell proliferation and apoptosis. Theriogenology.

[34] Fujibe Y., Baba T., Nagao S., Adachi S., Ikeda K., Morishita M., Kuno Y., Suzuki M., Mizuuchi M., Honnma H. (2019). Androgen potentiates the expression of FSH receptor and supports preantral follicle development in mice. J. Ovarian. Res..

[35] Dewailly D., Robin G., Peigne M., Decanter C., Pigny P., Catteau-Jonard S. (2016). Interactions between androgens, FSH, anti-Müllerian hormone and estradiol during folliculogenesis in the human normal and polycystic ovary. Hum. Reprod. Update.

[36] Okazaki K., Okazaki S., Nishimura S., Nakamura H., Kitamura Y., Hatayama K., Nakamura A., Tsuda T., Katsumata T., Nishikawa A. (2001). A repeated 28-day oral dose toxicity study of methoxychlor in rats, based on the ‘enhanced OECD test guideline 407’ for screening endocrine-disrupting chemicals. Arch. Toxicol..

[37] Tomic D., Frech M.S., Babus J.K., Gupta R.K., Furth P.A., Koos R.D., Flaws J.A. (2006). Methoxychlor induces atresia of antral follicles in ERalpha-overexpressing mice. Toxicol. Sci..

[38] Gervásio C.G., Bernuci M.P., Silva-de-Sá M.F., Rosa-E-Silva A.C. (2014). The role of androgen hormones in early follicular development. ISRN Obstet. Gynecol..

[39] Britt K.L., Findlay J.K. (2002). Estrogen actions in the ovary revisited. J. Endocrinol..

[40] Jayawardana B.C., Shimizu T., Nishimoto H., Kaneko E., Tetsuka M., Miyamoto A. (2006). Hormonal regulation of expression of growth differentiation factor-9 receptor type I and II genes in the bovine ovarian follicle. Reproduction.

[41] Hunzicker-Dunn M., Maizels E.T. (2006). FSH signaling pathways in immature granulosa cells that regulate target gene expression: Branching out from protein kinase A. Cell. Signal..

[42] Almeida F.R.C.L., Costermans N.G.J., Soede N.M., Bunschoten A., Keijer J., Kemp B., Teerds K.J. (2018). Presence of anti-Müllerian hormone (AMH) during follicular development in the porcine ovary. PLoS ONE.

[43] Abbott D.H., Padmanabhan V., Dumesic D.A. (2006). Contributions of androgen and estrogen to fetal programming of ovarian dysfunction. Reprod. Biol. Endocrinol..

[44] Quinn R.L., Shuttleworth G., Hunter M.G. (2004). Immunohistochemical localization of the bone morphogenetic protein receptors in the porcine ovary. J. Anat..

[45] Du P., Ye L., Li H., Ruge F., Yang Y., Jiang W.G. (2012). Growth differentiation factor-9 expression is inversely correlated with an aggressive behaviour in human bladder cancer cells. Int. J. Mol. Med..

[46] Du P., Ye L., Yang Y., Jiang W.G. (2014). Reduced expression of growth and differentiation factor-9 (GDF9) is associated with aggressive behaviour of human clear-cell renal cell carcinoma and poor patient survival. Anticancer Res..

[47] Durlej M., Knapczyk-Stwora K., Duda M., Galas J., Slomczynska M. (2011). The expression of FSH receptor (FSHR) in the neonatal porcine ovary and its regulation by flutamide. Reprod. Domest. Anim..

[48] Zhao S., Fernald R.D. (2005). Comprehensive algorithm for quantitative real-time polymerase chain reaction. J. Comput. Biol..

[49] Laemmli U.K. (1970). Cleavage of structural proteins during the assembly of the head of bacteriophage T4. Nature.

